# Mitochondrial defects in sporadic inclusion body myositis—causes and consequences

**DOI:** 10.3389/fcell.2024.1403463

**Published:** 2024-05-14

**Authors:** Elsie Chit Yu Iu, Ho So, Chi Bun Chan

**Affiliations:** ^1^ School of Biological Sciences, The University of Hong Kong, Pokfulam, Hong Kong SAR, China; ^2^ Department of Medicine and Therapeutics, Faculty of Medicine, The Chinese University of Hong Kong, Shatin, China

**Keywords:** mitochondria, myositis, muscle, pathogenesis, pyroptosis, necroptosis

## Abstract

Sporadic inclusion body myositis (sIBM) is a distinct subcategory of Idiopathic Inflammatory Myopathies (IIM), characterized by unique pathological features such as muscle inflammation, rimmed vacuoles, and protein aggregation within the myofibers. Although hyperactivation of the immune system is widely believed as the primary cause of IIM, it is debated whether non-immune tissue dysfunction might contribute to the disease’s onset as patients with sIBM are refractory to conventional immunosuppressant treatment. Moreover, the findings that mitochondrial dysfunction can elicit non-apoptotic programmed cell death and the subsequent immune response further support this hypothesis. Notably, abnormal mitochondrial structure and activities are more prominent in the muscle of sIBM than in other types of IIM, suggesting the presence of defective mitochondria might represent an overlooked contributor to the disease onset. The large-scale mitochondrial DNA deletion, aberrant protein aggregation, and slowed organelle turnover have provided mechanistic insights into the genesis of impaired mitochondria in sIBM. This article reviews the disease hallmarks of sIBM, the plausible contributors of mitochondrial damage in the sIBM muscle, and the immunological responses associated with mitochondrial perturbations. Additionally, the potential application of mitochondrial-targeted chemicals as a new treatment strategy to sIBM is explored and discussed.

## Introduction

Idiopathic inflammatory myopathies (IIM), commonly known as myositis, is a heterogeneous group of muscle diseases with a global prevalence of 2–25 individuals per 100, 000 persons (Khoo et al., 2023). Based on the clinical, serological, and histological examination results, IIM can be classified into 5 major subtypes: dermatomyositis (DM), polymyositis (PM), immune-mediated necrotizing myopathy (IMNM), overlap myositis (OM), and sporadic inclusion body myositis (sIBM). Despite each IIM subtype has distinctive clinical and unique histopathological features, a great majority of IIM patients suffer from muscle weakness with muscle edema, fatty infiltration in muscle fibers, and elevated serum creatine kinase (CK) level (Tsamis et al., 2022). Additionally, most IIM patients demonstrate overactivation of cellular and humoral immunity as revealed by the presence of infiltrating granzyme and perforin-secreting CD8^+^ cytotoxic T cells and B cells in muscle and autoantibodies in their serum (Goebels et al., 1996; Greenberg et al., 2005; Gunawardena et al., 2009). The involvement of innate immunity in IIM is also evidenced by the muscle enrichment of dendritic cells and M2 macrophages for tissue repair (Rinnenthal et al., 2014).

Because the muscles of IIM patients exhibit mild-to-severe inflammation phenotypes, most treatments for IIM target the uncontrolled immune response. Glucocorticoids (GC) are the front-line treatment that is widely adopted in PM and DM (Greenberg, 2019). They are often administrated with other immunosuppressants like azathioprine and methotrexate to reduce steroid-related side effects like osteoporosis and cardiovascular diseases (Oddis, 2016). Second-line treatments such as cyclosporine, tacrolimus, and intravenous immunoglobin are used to alleviate the disease symptoms in refractory or severe PM and DM cases (Zeng et al., 2022; Skolka and Naddaf, 2023). Nonetheless, these immunosuppressive agents are not enduringly effective in treating some IIM patients, particularly those with sIBM (Skolka and Naddaf, 2023). A recent study using human sIBM xenografts demonstrated that T cell depletion does not alleviate the muscle degenerative features (Britson et al., 2022), further challenging the pathogenic role of inflammation in the disease. Therefore, it is suspected that the immune response might not be the fundamental cause of muscle damage in sIBM. Because abnormal mitochondrial changes are commonly found in the muscle of sIBM patients, which are more prevalent than other IIM subtypes (Oldfors et al., 2006), impaired mitochondrial function might be an underestimated factor in the pathogenesis of sIBM. Several excellent reviews have discussed how mitochondrial dysfunction may generate muscle weakness in sIBM (De Paepe, 2019; Chapa et al., 2023; Danieli et al., 2023), but the underlying mechanism of mitochondrial defects remains inconclusive. In this article, we review the recent findings of mitochondrial dysfunction in sIBM and discuss their possible linkage with various disease symptoms. We will also discuss some potential mitochondrial-based therapeutic strategies for the treatment of sIBM.

## Symptoms, diagnosis, and current treatment of sIBM

sIBM is a male-predominant disease that has a prevalence of 5–71 per million in the whole population (Tsamis et al., 2022). Typically occurring after the age of 50 (Snedden et al., 2022), sIBM is characterized by progressive and asymmetric weakness in the quadriceps and long finger flexors rather than an acute or subacute symmetrical proximal muscle weakness seen in other subtypes of IIM (Zubair et al., 2023). Individuals with sIBM experience a loss of muscle strength at a rate of 3.5%–28% per year (Zubair et al., 2023) and can become wheelchair-bound at a median time of 10.5 years after the disease onset (Skolka and Naddaf, 2023). Respiratory compromise and dysphagia are common risk factors for premature death in sIBM patients, which may account for their slight but significantly shortened lifespan when compared to healthy subjects (Shelly et al., 2021). Recently, an association between sIBM and malignancy has been proposed, although sIBM is not generally regarded as a risk factor for cancer (Damian et al., 2022). Due to the slowly progressive nature of symptom development, there is often a delay of 5–10 years between disease onset and confirmation (Huntley et al., 2019).

The diagnosis of sIBM is typically made in patients having experienced disease symptoms for more than 12 months with onset after 45-year-old, (Greenberg, 2019; Skolka and Naddaf, 2023), and most importantly exhibit histopathological hallmarks in their muscle, including endomysial lymphocyte infiltration, rimmed vacuoles, protein deposition, ragged red fibres, and the presence of cytochrome c oxidase (COX)-negative myofibers (Tanboon et al., 2020). The accumulation of proteins like amyloid beta (Aβ), ubiquitin, phosphor-tau, sequestosome 1 (p62), and TAR DNA binding protein 43 kDa (TDP-43) is also detected in the muscle of some sIBM patients (Skolka and Naddaf, 2023). Autoantibodies against cytosolic 50-nucleotidase (cN-1A), an enzyme highly expressed in the skeletal muscle that catalyses the hydrolysis of adenosine monophosphate (AMP) into adenosine and inorganic phosphate (Salam et al., 2022; Diederichsen et al., 2023) can be found in many sIBM patients but not in any other IIM subtypes (Yamashita et al., 2023), making it an important evidence to the disease diagnosis. Nevertheless, a substantial number of sIBM patients displayed no elevated anti-cN-1A antibody in their circulation, suggesting that the presence of antibody should not be considered a necessary criterion in the disease diagnosis (Mavroudis et al., 2021; Diederichsen et al., 2023). While histological analysis of muscle biopsy remains an essential procedure for sIBM diagnosis (Papadimas et al., 2019; Winkler et al., 2021), other methods such as multi-osmics profiling are being developed to screen for novel biomarkers with 100% sensitivity and specificity (Cantó Santos et al., 2023).

The presence of anti-cN-1A antibodies and the elevated level of inflammatory cytokines like interferon γ (IFN-γ), tumor necrosis factor α (TNF-α), interleukin 7 (IL-7), and interleukin 32 (IL-32) in the serum of sIBM patients indicate that both humoral and cellular immunities are provoked (Tsamis et al., 2022). The hyperactivation of cellular immunity in sIBM is demonstrated by the transformation of CD4^+^ helper T-cells into CD28^−^ cytotoxic T-cells and the infiltration of CD8^+^ T cells in the patient’s muscle (Miller et al., 2018). These cytotoxic T-cells might upregulate the muscular expression of major histocompatibility complex I (MHC-I) genes and induce muscle cell death, possibly via perforin-granzymes or Fas ligand-mediated mechanisms (Fyhr and Oldfors, 1998; Snedden et al., 2022). Indeed, immunohistochemical analysis revealed stronger Fas immunoreactivity in the atrophic fibres of sIBM patients than that in other IIM subtypes (De Bleecker et al., 2001). Despite the clear engagement of the immune system in the pathogenesis of sIBM, disease alleviation via immune response inhibition is not satisfactory, as a significant number of patients are irresponsive to the classical immunosuppressive treatment (Askanas and Engel, 2008; Rygiel et al., 2015). For instance, some sIBM patients receiving long-term GC treatment for more than 5 years had a similar number of T-cells-invaded myofibers as those patients without treatment (Pruitt et al., 1996). Muscle atrophy of sIBM patients has also not ceased even though their CK levels have returned to normal after GC treatment (Miller et al., 2018). Moreover, the extent of immune cell infiltration in sIBM patients was found to correlate poorly with the severity of muscle weakness (Lightfoot et al., 2015). Hence, it is argued that prolonged immune system activation in sIBM is secondary to the intrinsic defects in the skeletal muscle.

## Presence of abnormal mitochondria in sIBM

### Mitochondrial DNA (mtDNA) mutation and deletion

A distinguishing characteristic of sIBM that differentiates it from other IIM subtypes is the presence of ragged red fibers in the patient’s muscle (Boncompagni et al., 2012; Tanboon et al., 2020), which appear as red rim in the speckled sarcoplasm after Gomori Trichrome staining. Because ragged red signal is caused by the accumulation of defective mitochondria below the plasma membrane, these unusual histological signals are not exclusive to sIBM but are also seen in the muscle of patients suffering from primary mitochondrial disorders, such as myoclonic epilepsy with ragged red fibers (MERRF) and mitochondrial thymidine kinase (T2K) deficiency (DiMauro et al., 1985; Jou et al., 2022). Indeed, some mitochondria in the muscles of sIBM patients are aberrant in structure and function. In contrast to DM, where single point mutations in mtDNA are frequently detected (Zhao et al., 2022), sIBM muscle features large-scale, single segment deletions (i.e., major rearrangement mutation) in the major arc of mtDNA molecules. It has been reported that 122 deletion breakpoints and 33 different single nucleotide deletions were detected in the mtDNA molecules of sIBM patients (Moslemi et al., 1997), which could lead to the elimination of up to 1/3 of the whole mtDNA. Another study confirmed an average of 67% lower mtDNA copy number in the quadriceps and tibialis anterior muscle of sIBM patients than in healthy subjects (Bhatt et al., 2019). Interestingly, heterogenous mtDNA molecules with differential deletion regions could be detected in a single myofiber (Rygiel et al., 2016), resulting in a high heteroplasmy in the muscle. Moreover, the mtDNA deletion might be present in continuous segments of the same muscle fiber, creating a spatially unique pattern of the mitochondrial protein COX staining within a single myofiber (Oldfors et al., 1995; Horvath et al., 1998).

### Biochemical outcomes of mtDNA deletion in sIBM—oxidative phosphorylation (OXPHOS) defects

Because the human mtDNA encodes genes for electron transfer complexes (ETC) subunits, mtDNA deletion might result in the loss of mitochondrial content (Joshi et al., 2014; Catalán-García et al., 2016a; Oikawa et al., 2020; Hedberg-Oldfors et al., 2021). In fact, the severity of mtDNA deletion is highly associated with the number of COX-deficient fibers, which are myofibers that contain abnormally low levels of the mitochondrial complex IV, in the sIBM patients’ muscles (Landfeldt et al., 2015). Using single-cell analysis, Rygiel reported that ∼85% of COX-deficient cells have major mtDNA deletion and rearrangement (Rygiel et al., 2016). Mitochondrion morphology is also disrupted in the myofiber of sIBM patients, with shortened and enlarged cristae and junction breaks, resulting in a reduced mitochondrial length/width ratio (Oikawa et al., 2020). As COX is an essential component of the ETC for ATP synthesis via OXPHOS, COX deficiency in the myoblasts of sIBM patients leads to a shift in ATP production from OXPHOS to glycolysis (Oikawa et al., 2020), which is a key indicator of mitochondrial dysfunction (Di Leo et al., 2023). The ^31^P-magnetic resonance spectroscopy assessments revealed that the muscle of sIBM patients has a low ability to synthesize ATP during resting, further supporting the notion of impaired mitochondrial respiration in sIBM (Lodi et al., 1998). A recent study also reported diminished mitochondrial enzymatic activities in cultured sIBM myofibers under low glucose availability, indicating compromised metabolic flexibility (Catalán-García et al., 2020). Consequently, this low mitochondrial activity might significantly impact the overall function of skeletal muscle, causing weakness in contraction strength and endurance (Gonzalez-Chapa et al., 2023).

### Potential causes of mtDNA deletion in sIBM—error in mtDNA replication

The occurrence of mtDNA deletion in the muscle of sIBM patients is not limited to a consensus locus within a mtDNA molecule but is present in multiple regions. This suggests that the cause of mtDNA deletion and rearrangement might be more complex than previously assumed. The underlying mechanism that leads to the high frequency of mtDNA deletion in sIBM is still unknown. However, it has been hypothesized that most truncated mtDNA molecules in sIBM muscle are generated from the clonal expansion of a single defective molecular species (Moslemi et al., 1997). It has also been suggested that the mtDNA replication process in sIBM muscle is prone to errors, further contributing to the generation of multiple copies of defective mtDNA (Horvath et al., 1998). The high replication error in sIBM is not caused by any genetic defects in nuclear genes engaged in mitochondrial genome maintenance, as a recent study found no pathogenic variants in nuclear genes that contribute to the high levels of mtDNA deletions in sIBM (Hedberg-Oldfors et al., 2021). Instead, single nucleotide polymorphism of several key genes involved in mtDNA replication and maintenance, including the DNA helicase Twinkle, the DNA polymerase γ (POLG), and ribonucleotide-diphosphate reductase subunit M2B (RRM2B), has been identified in sIBM patients (Lindgren et al., 2015). Nevertheless, the functional consequence of these single mutations has not been elucidated.

### Potential causes of mtDNA deletion in sIBM—reactive oxygen species (ROS) accumulation

A possible cause of high mtDNA damage in sIBM muscle is its unique localization and structure. Mitochondria are the organelle that produces ∼90% of the total ROS in the cells as the byproducts of, ETC complex I and III (Favaro et al., 2019). These short-lived yet highly reactive molecules are unstable and cause structural damage to mtDNA molecules (Urbina-Varela et al., 2020). The histone-free nature of mtDNA molecules further increases their risk of ROS-induced damage (Taylor and Turnbull, 2005). Histones protect DNA against hydroxyl radical-induced strand breaks, which is a critical defense mechanism against DNA damage (Ljungman and Hanawalt, 1992). Indeed, the high ROS level and mitochondrial deletion are closely associated with each other in many diseases, such as hepatocellular carcinoma (Moriya et al., 2001) and chronic periodontitis (Canakçi et al., 2006). It is important to note that this “ROS-induced mutation” model can only be valid if the ROS content in the muscle of sIBM patients is higher than that of the healthy subjects. Several studies have demonstrated that ROS level is augmented in the muscles of sIBM. First, myoblasts of sIBM patients displayed a higher ROS concentration when treated with a glutathione synthesis inhibitor (Oikawa et al., 2020). Second, a high level of deglycase DJ-1, an important mitochondrial protective protein against oxidative stress (Taira et al., 2004), has been found in the mitochondria of sIBM muscle (Terracciano et al., 2008), indicating the mitochondria are under high oxidative stress and require stronger protection. In support of this notion, fibroblasts cultured from sIBM patients displayed higher oxidative stress, concomitant with increased antioxidant defense (Cantó-Santos et al., 2023). Furthermore, the protein amount and gene expression of ROS scavengers, Cu, Zn- superoxide dismutase (Cu, Zn-SOD) and manganese superoxide dismutase (Mn-SOD), are augmented in vacuolated myofibers in sIBM (Askanas and Engel, 1998; Tsuruta et al., 2002). However, comparable muscular lipid peroxidation between sIBM patients and healthy subjects was also reported (Catalán-García et al., 2016b), making the ROS hypothesis inconclusive. Instead of having more mutation inducers in the muscle, it is possible that the DNA repair system is blemished in the patient’s tissue, hence facilitating the propagation of mutated mtDNA. Because no examination of the activity of DNA repairing machinery, such as apurinic/apyrimidinic endonuclease (APE) or DNA damage-binding protein (DDB) (Jang et al., 2019), in the sIBM sample, has been performed, it remains unknown if the system is involved in the accumulation of truncated mtDNA.

### Potential causes of mtDNA deletion in sIBM—β amyloid (Aβ) overproduction

In addition to the occurrence of mtDNA deletion, which resulted in a loss of mitochondria in the muscle (Hedberg-Oldfors et al., 2021), the accumulation of Aβ in the tissue might further impair the function of the existing mitochondria in sIBM. Studies have shown that overproduction of β amyloid precursor protein (APP) in human muscle fibers resulted in decreased COX activity, enlarged mitochondria, and the formation of disrupted cristae that resemble the pathological features of sIBM (Askanas et al., 1996). Abnormal mitochondrial-related functions, including increased rate of ROS production, reduced TCA cycle activities, and a shift of fatty acid-to-glucose utilization, were also seen in the muscle of APP transgenic mice (Boncompagni et al., 2012). It is believed that the mislocation of Aβ to the mitochondrial membrane impedes the function of the mitochondrial transporter, hence hindering the imports of materials that are indispensable for mitochondrial functions (Askanas et al., 1996; Devi et al., 2006). Although this Aβ-mitochondrial interaction, as observed in neuronal tissues, is a logical linkage of Aβ overproduction to the dysregulated mitochondrial function, no colocalization of Aβ and mitochondria in the muscle of sIBM patients has been reported.

### Potential causes of mtDNA deletion in sIBM—defective autophagy

It is also possible that the mitochondrial defect in sIBM muscle is attributed to the delayed organelle turnover, which might result in the buildup of dysfunctional mitochondria. The “dysregulated myoproteostasis” model, which covers protein synthesis defect, improper folding, extensive post-translational modification, and impaired degradation of proteins, has been proposed by Askanas et al. to collectively explain the aggregation of abnormal proteins and organelles in sIBM muscle (Askanas et al., 2015). Several studies report that organelle degradation by macroautophagy is compromised in sIBM. First, there is a lack of p62 binding accuracy to LC3 in the patient’s muscle, although sIBM muscles contain a higher frequency of LC3-positive autophagosomes, indicating a stop of the autophagy process in its initial stages (Lünemann et al., 2007; Nogalska et al., 2010; Suzuki et al., 2019). Moreover, the activity of lysosomal enzymatic activity cathepsin D and B was lower than the healthy control in the sIBM muscle, which delays the clearance of LC3-associated autophagosomes and the ubiquitinated proteins (Nogalska et al., 2010). Indeed, the muscle biopsies of sIBM patients showed a high density of lipofuscin aggregates, a marker of lysosomal dysfunction (Lu et al., 2020). Nicot et al. (2014) further specified the mechanism by demonstrating that the autophagy cargo receptor NBR1-mediated removal of protein aggregates was inhibited in sIBM. This delayed autophagy provides a logical explanation for the accumulation of p62 and Aβ aggregates in the autophagic vacuoles in sIBM (Güttsches et al., 2017). Furthermore, several genes associated with autophagosome-lysosome processing have been identified as the risk alleles in sIBM (Weihl et al., 2015; Gang et al., 2016; Papadopoulos et al., 2017). Based on these findings, pharmacological inhibition of autophagy by chronic colchicine administration in mice is used as an animal model for sIBM research (Ching et al., 2013). Interestingly, chaperone-mediated autophagy is increased in the muscle of sIBM, as evidenced by elevated levels of lysosomal membrane protein LAMP2A and the chaperone Hsp70 co-aggregates (Cacciottolo et al., 2013). Because chaperone-mediated autophagy mainly targets specific proteins but not large organelles, this escalated chaperone-mediated autophagy might be a compensatory response to abnormal protein aggregation, like p62, in sIBM. Given that the removal of damaged mitochondria is autophagy-dependent, the dysregulated autophagy in sIBM might jeopardize its clearance. Although no comprehensive assessment of mitophagy, the specific pathway of autophagy to induce mitochondria degradation (Lu et al., 2023), has been performed in sIBM muscle, decreased expression of dynamin-related protein 1 (DRP1) has been detected in cultured myoblasts of sIBM patients (Oikawa et al., 2020). DRP1 is a critical factor that promotes the splitting of mitochondria for subsequent degradation (Picca et al., 2023); this low level of DRP1 indirectly supports the hypothesis that the clearance of mitochondria in sIBM muscle might be impaired. Moreover, abnormal accumulation of the mitochondrial fusion marker mitofusin 1 (MFN1) and the mitophagy receptor Bcl-2 adenovirus E1B19 19-kDa interacting protein (BNIP3) was observed in the ragged red fibers of the sIBM patients (Askanas et al., 2015). Because mitofusin accumulation triggers mitochondrial enlargement, which hinders the sequestration of damaged mitochondria (Joaquim and Escobar-Henriques, 2020), augmented mitofusin content in the sIBM muscle may result in defective clearance of Bnip3-tagged cargos.

## Mitochondrial defects and immune response in muscle

Although the invasion of immune cells is commonly observed in all IIM, inflation of highly differentiated CD8^+^CD28^−^ cytotoxic T cells is uniquely present in sIBM (Greenberg and Bourc’his, 2019). It is proposed that autoimmunity is the root cause of sIBM pathogenesis, as genetic mutation of TDP-43 and p62 in myotilinopathies and desminopathies do not result in the formation of protein aggregates (Olivé et al., 2008; Olivé et al., 2009). Moreover, stimulation of cultured muscle cells by inflammatory cytokines, such as IL-1β and IFNγ, or upregulation of much MHC class I alone is sufficient to trigger the formation of protein aggregates and rimmed vacuoles (Fréret et al., 2013; McCord and Day, 2023a; McCord and Day, 2023b). Furthermore, rhabdomyosarcoma cells challenged with the IgG from sIBM patients effectively induced the aggregation of p62 (Tawara et al., 2017). Finally, altering the immune system by virus infection like HIV can produce pathological features of sIBM like p62 aggregates and the formation of rimmed vacuoles in the muscle (Hiniker et al., 2016). While these findings support the causal relationship between autoimmunity and muscle degeneration, mitochondrial defects in the muscle of sIBM might have feed-forward activity to exaggerate tissue inflammation. Supporting this notion, Shelton et al. (2019) found that mutation of the mitochondrial transporter, aspartate glutamate carrier 1 (AGC1), produced a proinflammatory phenotype in the muscle biopsies of dogs. Indeed, recent studies in mitochondrial biology have confirmed that excessive mitochondrial dysfunction can trigger tissue damage and subsequent immune responses via regulated cell death mechanisms.

In principle, damaged mitochondria release their organelle contents, such as mtDNA, cardiolipins, and Ca^2+^, into the cytosol and the extracellular space (Picca et al., 2023). These mitochondrial-derived damage-associated molecular patterns (DAMPs) are regarded as foreign molecules by the pattern recognition receptors of the immune cells due to the bacterial ancestry of mitochondria. Induction of the innate immune response triggers the activation of pro-inflammatory pathways like toll-like receptor (TLR) signalling (Picca et al., 2023). Indeed, it has been reported that binding of oxidized cardiolipins to TLR4 in the cytosol initiates the NF-κB signalling, leading to increased myostatin (MSTN) expression in the sIBM muscle (Sachdev et al., 2018).

The presence of mtDNA in the cytosol is an intrinsic warning of pathogen infection or cellular dysfunction. These cytosolic mtDNAs are sensed by the cyclic guanosine monophosphate (GMP)-AMP synthase (cGAS) and promote its dimerization, leading to the production of second messenger cyclic GMP-AMP (cGAMP). The binding of cGAMP to the endoplasmic reticulum protein STING (stimulator of interferon genes) induces its translocation to the Golgi apparatus, where it activates the TANK-binding kinase 1 (TBK1) to phosphorylate the transcription factors interferon regulatory factor 1 (IRF1) and the IκB kinase complex. Consequently, transcription of NFκB-targeted genes such as type I interferons will be enhanced, attracting the immune cells to the injured muscle (Zhang et al., 2022). Although no studies have been performed to evaluate the cGAS-STING pathway in the animal models of sIBM or patient samples, the mtDNA-induced cGAS-STING pathway activation is detected in cells when TDP-43 invades mitochondria (Yu et al., 2020). Moreover, a recent report by Irazoki et al. (2023) demonstrated that the release of mtDNA after mitochondrial dysfunction is sufficient to induce sterile inflammation in the skeletal muscle, further supporting the role of cGAS pathway in myositis development.

The release of mtDNA may activate the pyroptotic cell-death pathways via activating another intracellular DNA sensor NLRP (nucleotide-binding oligomerization domain, leucine rich repeat and pyrin domain containing) proteins to form inflammasomes (Marchi et al., 2023) and the subsequent pyroptosis signalling. Inflammasomes are multiprotein complex containing leucine-rich repeated containing proteins (e.g., NLRP2, AIM2, Pyrin), the adapter protein ASC (apoptosis-associated speck-like protein containing a caspase recruitment domain CARD), and pro-caspase 1. Although the molecular details of mtDNA-induced NLRP activation are still unknown, the outcomes of inflammasome formation have been well-defined. Once the caspase 1 in the inflammasome is activated, it cleaves the membrane pore protein Gasdermin D and interleukins (IL-1 and IL-8). Consequently, the membrane permeability is increased, leading to cell swelling, leakage of cellular proteins, and the formation of functional IL-1β and IL-18, all of which are strong inflammation inducers (Sharma and Kanneganti, 2021). A recent study demonstrated that the formation of NLRP3 inflammasome and pyroptosis was upregulated in the muscle fibers of DM and PM (Liu et al., 2021), its activity in sIBM remains to be investigated. Nevertheless, it has been demonstrated that ketogenic diet could alleviate the clinical symptoms of sIBM patients, possibly through suppressing NLRP3 inflammation activation, suggesting that pyroptosis is involved in the pathogenesis of sIBM (Phillips et al., 2020).

Recently, Kamiya et al. (2022) reported that inhibiting the necroptosis signalling effectively ameliorated the invasion of CD8^+^ cytotoxic T lymphocytes and thus suppressed muscle injury in PM. Necroptosis is a caspase-independent form of programmed cell death that results in membrane rupture, cell swelling, and leakage of DAMPs to promote inflammation (Pasparakis and Vandenabeele, 2015). The canonical necroptosis pathway is typically initiated by the cytokine TNFα in caspase 8-inactive cells. Once TNFα binds to its cognate receptor TNFR1, the receptor forms a complex that contains TNFR1-associated death domain protein (TRADD) and receptor-interacting serine/threonine protein kinase 1 (RIPK1). In certain conditions such as growth factor deprivation, the ligand activated TNFR-TRADD-RIPK1 complex further recruits and activates caspase 8 to cleave the downstream apoptosis executors like BH3-interacting domain death agonist (BID) to induce mitochondrial member depolarization and release of cytochrome c for caspase 3 activation (Luo et al., 1998). In cells with the absence of caspase 8, activation of TNFR1 promotes the heterodimerization of RIPK1 and RIPK3, forming the complex necrosome. Because RIPK3 is a proteolytic substrate of caspase 8, the formation of necrosome will only be formed in the absence of caspase 8 (Kang et al., 2013). The necrosome further recruits the mixed-lineage kinase domain-like protein (MLKL), which is phosphorylated by RIPK3 to form active oligomers for plasma membrane translocation. Due to its porous nature, the insertion of MLKL oligomers causes leakage of cellular content to the extracellular environment or cell rupture to induce the tethering of immune cells. Interestingly, activation of RIPK1 and RIPK3 triggers mitochondrial dysfunction (Chen et al., 2018) and activates the pyruvate dehydrogenase complex to produce excessive ROS (Yang et al., 2018), respectively. The high ROS feedbacks to the necrosome complex formation, forming a positive feedback loop of the necroptosis pathway to further enhance the necroptosis signalling (Qiu et al., 2018). Peng et al. (2022) reported that the necroptosis machinery is highly expressed in several subtypes of IIM, including DM and IMNM. Moreover, overactivation of the necroptotic pathway is sufficient to cause cell death of healthy muscle cells (Peng et al., 2022). Although high expression of necroptosis inducer TNF-α is detected in the muscle of sIBM patients (Schmidt et al., 2008), the activity of caspase 8 in the muscle has never been studied, making it a mystery if necroptosis is also elevated in sIBM like other IIM subtypes.

## Targeting mitochondrial health as a new treatment strategy of sIBM

Due to the immunosuppressant-resistant feature of sIBM, exercise remains the main-stay therapy for sIBM (Koo et al., 2019). In general, endurance exercise is known to benefit skeletal muscle by increasing the disposal of ROS-damaged proteins and the synthesis of new mitochondria to accelerate mitochondrial turnover, thereby maintaining a healthy mitochondria network with boosted OXPHOS capacity (Sorriento et al., 2021). Resistance exercise also enhances the cellular antioxidation capacity by activating FOXO3, a transcription factor that induces the expression of antioxidant enzymes like SOD, to improve muscle function in sIBM (Koo et al., 2019). Interestingly, Coudert et al. (2022) recently reported that testosterone supplementation and exercise training may exert additive effects in improving muscle performance and mitigating the overactivated immunity in sIBM patients. However, performing regular exercise relies heavily on self-motivation and is difficult for cane- or wheelchair-bound patients at the advanced stage of IBM. Therefore, there is an urge to develop agents that recapitulate the benefits of exercise, such as upregulating antioxidant activity, increasing mitochondrial biogenesis, or promoting mitochondrial removal in the muscle. Considering that AMPK activation is a critical event in initiating mitochondrial biogenesis and mitophagy during exercise, AMPK-activating agents are attractive candidates for this purpose. Indeed, the AMP analogue 5-Aminoimidazole-4-carboxamide-1-beta-D-ribofuranosyl 5′-monophosphate (AICAR) downregulated the expression of atrophic marker Atrogin/MAFbx in the gastrocnemius muscle and relieved cancer-induced muscle atrophy in mice (Hall et al., 2018). Another Food and Drug Administration (FDA)-approved AMPK activator, metformin, is found to promote mitophagy in type 2 diabetic patients (Picca et al., 2023). In a cardiovascular disease mouse model, metformin treatment suppressed ROS production in the abnormal heart tissue in an AMPK-dependent manner, which prevented the NLRP3/IL-1β-mediated inflammation and cardiovascular lesions (Marek-Iannucci et al., 2021).

Antioxidant application may be helpful in rescuing cell death in the sIBM muscle by neutralizing ROS into less harmful products (Johnson et al., 2023). For example, the well-known antioxidant polyphenol resveratrol has been shown to suppress myostatin-mediated cell death by activating the NF-κB signalling in cultured sIBM myoblasts (Askanas et al., 2012). The use of mitochondrial-targeted antioxidants like MitoQ and MitoVitE, which are hundred folds more effective in rescuing mitochondrial oxidative stress-induced cell death in human fibroblasts than other non-specific, cellular antioxidants like idebenone and vitamin E (Jauslin et al., 2003), might also alleviate the symptoms of sIBM (Rostamzadeh et al., 2024). Indeed, studies in numerous experimental models have confirmed the protective effect of these mitochondrial-targeted antioxidants in treating ROS-associated diseases like Parkinson’s Disease and atherosclerosis (Jiang et al., 2020; Sulaimon et al., 2022), which provide a solid scientific basis to extend their translational potential in ameliorating sIBM. Nevertheless, caution must be exercised when antioxidants are used in the sIBM treatment because exogenous supplementation of NADH and GSH may disrupt the redox balance and impose reductive stress on the cell (Xiao and Loscalzo, 2019). In fact, multiple studies have correlated excessive reductive stress with the development of inflammatory-associated diseases like cardiomyopathy, muscular dystrophy, and Alzheimer’s disease (Pérez-Torres et al., 2017).

Target mitophagy, a specific form of autophagy that degrades mitochondria exclusively (Lu et al., 2023), may also be a viable approach to alleviate myopathies associated with excessive oxidative stress (Ito et al., 2022). Traditional mitophagy inducers like oligomycin and carbonyl cyanide m-chlorophenyl hydrazone (CCCP) have been widely adopted in the *in vitro* system but their high toxicity limits their clinical applicability (Lee et al., 2023). Therefore, considerable efforts have been devoted to screen for alternative inducers with low toxicity for clinical applications. In 2021, Luan et al. (2021) reported that Urolithin A (UroA) reversed the declined mitophagy in cultured myoblasts of Duchenne Muscular Dystrophy (DMD) patients and *mdx* mice, which led to a reduction in muscle damage. UroA is a metabolite of microflora produced from the polyphenols ellagic acid and ellagitannins in food (Faitg et al., 2024), which is considered safe for oral consumption in humans as a dietary supplement by the FDA (García-Villalba et al., 2022). In a randomized, double-blinded, placebo-controlled clinical trial, subjects consuming UroA for 4 months exhibited augmented expression of mitophagy markers in their muscle biopsy, which was associated with higher complex I and II-mediated respiration (Faitg et al., 2024). Another clinical trial demonstrated that UroA effectively improved mitochondrial function in the skeletal muscle of the elderly by upregulating mitochondrial gene expression (Andreux et al., 2019). The positive effect of UroA on mitochondrial respiration was also reflected by the 65% higher running capacity in the UroA-administrated Wistar rats (Nat Med, 2016). Mechanically, UroA triggers mitophagy via lowering the mitochondrial membrane potential, as short-term UroA treatment induced membrane depolarization followed by augmented mitophagy marker expression (Ryu et al., 2016). Based on the promising effect of UroA in improving muscle function of age-related atrophic fibers, which share some biochemical characteristics of sIBM myofibers (Romani et al., 2021), it is reasonable to assume that UroA could also be effective in ameliorating mitochondrial defects and reducing muscle damage in sIBM. In supporting the idea that maintaining mitochondria activity is important to sustain the survival of the sIBM muscle cells, Oikawa et al. (2020) reported that mitochonic acid 5 (MA-5), a plant derivative that facilitates mitochondrial activities, such as increasing ATP synthesis, reducing ROS production, and promoting OXPHOS, was effective in protecting the myoblasts of sIBM patients from the buthionine sulfoximine-induced cell death. Presumably, mitochondrial health-improving agents like UroA and MA-5 might represent safe and effective agents for sIBM patients to improve their muscle function.

## Conclusion

**FIGURE 1:**
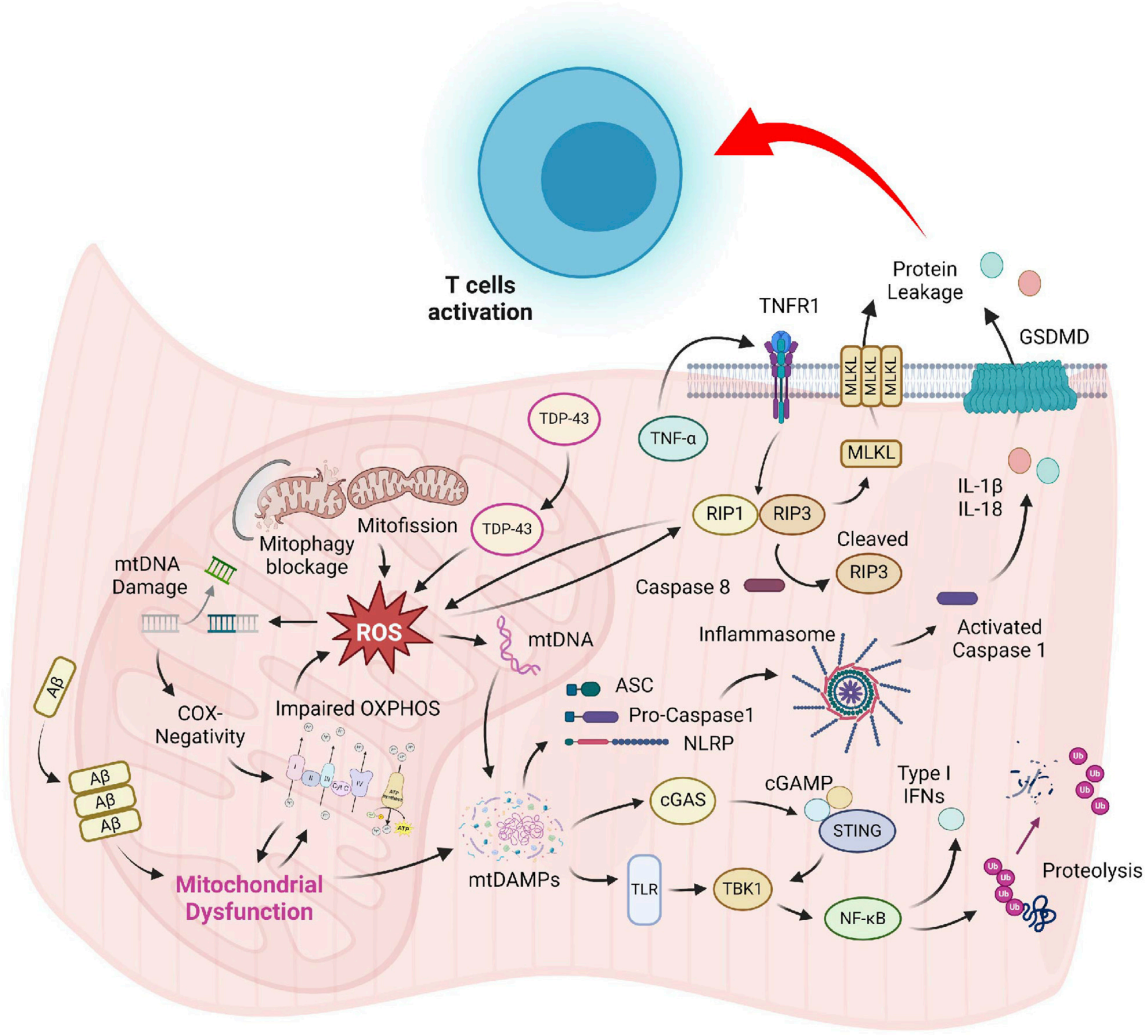
Prominent mitochondrial abnormalities in sIBM muscle include COX-negativity, OXPHOS suppression, delayed organelle clearance, and mislocalization of proteins. Augmented ROS produced by the dysfunctional mitochondrial might damage the organelle, resulting in the release of mitochondrial content into the sarcoplasm. These mtDAMPs are strong inducers of the TLR and cGAS-STING pathway, which promote inflammatory cytokines production and muscle breakdown via the NF-κB signaling. Accumulation of mtDAMPs and ROS might also induce the formation of NLRP-mediated inflammasome and TNF-α-triggered necrosome, leading to compromised sarcolemma integrity. Leakage of cellular content generates DAMPs that might serve as activation signals for T-cell recruitment in sIBM.

Although pathological hallmarks of sIBM have been recognized for several decades, the precise molecular mechanism for these cellular abnormalities is still mysterious. Most studies on sIBM pathogenesis are associative in nature, and the lack of mechanistic studies hinders the development of effective treatment, making it still an incurable disease nowadays. The slow progress in the development of new sIBM therapy can be attributed, in part, to the uncertainty of the sequential relationship between muscle damage and overactivated immune response. Moreover, most studies of sIBM have primarily focused on the detrimental consequence of hyperactive immune cells and protein aggregation on skeletal muscle; the outcomes of other damaged organelles like mitochondria have received little attention. The conventional view of mitochondria solely as ATP-producing powerhouses biased our perception that defective mitochondria in IIM might only result in metabolic deficiency, thus underestimating its functional outcomes. Recent discoveries that highlight mitochondrial defects as inducers of immune response via pyroptosis and necroptosis in many different issues have provided new insights into the pathogenesis of IIM (Figure 1). Hence, it is imperative to recognize the etiological role of mitochondria and developing novel drugs that improve the mitochondrial health as a novel treatment strategy for sIBM.
